# Disrupting the TRAF1/cIAP2 interaction attenuates inflammasome activation and protects against monosodium urate crystal–induced arthritis

**DOI:** 10.1093/immhor/vlaf065

**Published:** 2025-11-24

**Authors:** Sahib Singh Madahar, Ali Mirzaesmaeili, Jonathan Raspanti, Yitian Tang, Ali A Abdul-Sater

**Affiliations:** Department of Biology, York University, Toronto, ON, Canada; School of Kinesiology and Health Science, Muscle Health Research Centre, York University, Toronto, ON, Canada; School of Kinesiology and Health Science, Muscle Health Research Centre, York University, Toronto, ON, Canada; School of Kinesiology and Health Science, Muscle Health Research Centre, York University, Toronto, ON, Canada; School of Kinesiology and Health Science, Muscle Health Research Centre, York University, Toronto, ON, Canada; Department of Biology, York University, Toronto, ON, Canada; School of Kinesiology and Health Science, Muscle Health Research Centre, York University, Toronto, ON, Canada

**Keywords:** cIAP2, crystal-induced arthritis, inflammasomes, TRAF1, ubiquitination

## Abstract

Tumor necrosis factor receptor (TNFR)–associated factor 1 (TRAF1) regulates NF-κB signaling and is implicated in chronic autoimmune diseases characterized by persistent inflammation. In addition to its role in restraining linear ubiquitin assembly complex–mediated linear ubiquitination of apoptosis-associated speck-like protein containing a caspase recruitment domain (ASC) to limit inflammasome activation, TRAF1 also stabilizes cellular inhibitor of apoptosis protein 2 (cIAP2) by protecting it from degradation. Notably, cIAP2 promotes inflammasome activation via K63-linked polyubiquitination of caspase-1. Here, we show that disrupting the TRAF1/cIAP2 interaction (V203A in humans; V196A in mice) reduces inflammasome activation. TRAF1^V203A^ THP-1 cells exhibit diminished caspase-1 ubiquitination, leading to impaired IL-1β secretion. Similarly, TRAF1^V196A^ mice produce significantly lower IL-1β levels after LPS challenge. In a monosodium urate crystal–induced arthritis model, TRAF1^V196A^ mice show reduced joint inflammation, decreased synovial immune cell infiltration, and attenuated disease severity. These findings establish the TRAF1/cIAP2 axis as a key regulator of inflammasome activation and a potential therapeutic target for inflammasome-driven diseases such as gout.

## Graphical abstract

**Figure:**
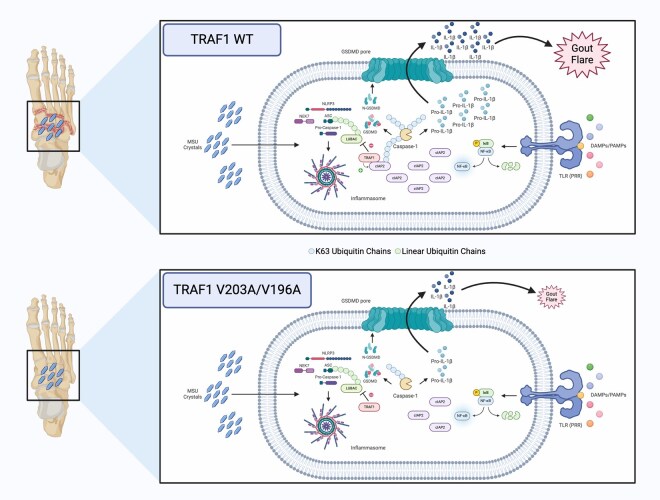

Key Findings Disrupting TRAF1/cIAP2 interaction impairs caspase-1 ubiquitination, reducing inflammasome activation and IL-1β secretion. TRAF1^V196A^ mice produce less IL-1β after LPS challenge, confirming its role in inflammasome regulation. TRAF1^V196A^ mice have reduced joint inflammation in a monosodium urate crystal–induced arthritis model.

## Introduction

Gout (or crystal arthritis), the most common type of inflammatory arthritis globally, is characterized by hyperuricemia (serum urate levels >7 mg/mL) and reflects the cardinal signs of inflammation.^1–5^ Mechanistically, the deposition of monosodium urate (MSU) crystals in the joints is sensed by macrophages and neutrophils. This leads to the activation of signaling cascades that promote inflammation and assemble inflammasome complexes, such as the NOD-like receptor (NLR) family pyrin domain–containing protein 3 (NLRP3).^6^^,^^7^ The activation of the NLRP3 inflammasome requires two signals: the priming signal (signal 1) comes from pathogen-associated molecular patterns (eg lipopolysaccharide [LPS]), and the activation signal (signal 2) includes a multitude of danger-associated molecular patterns (eg MSU crystals, extracellular ATP, or the bacterial toxin nigericin).^8–11^ The culmination of these two signals ultimately leads to the cleavage of pro-caspase-1 to activate caspase-1. This is then followed by the proteolytic cleavage of pro-IL-1β and pro-IL-18 into their biologically active forms, IL-1β and IL-18, by caspase-1. Interestingly, caspase-1 has been recently identified as an important player in the formation of gasdermin D (GSDMD) membrane pores that permit the release of these proinflammatory cytokines from cells.^3^^,^^7^^,^^12–14^ Caspase-1 and caspase-4/5/11 have also been implicated in inducing pyroptosis by cleaving the linker between the gasdermin-N and gasdermin-C domains in GSDMD and thereby relieving the intramolecular inhibition on the gasdermin-N domain.^15^^,^^16^

Dysregulation of inflammasomes can contribute to persistent and uncontrolled inflammation that characterizes inflammatory arthritis. Fortunately, various posttranslational modifications (PTMs) have been identified that regulate the activation of the well-studied NLRP3 inflammasome at multiple levels, including NLRP3, apoptosis-associated speck-like protein containing a caspase recruitment domain (ASC), and caspase-1. These PTMs include ubiquitination, phosphorylation, SUMOylation, and S-nitrosylation.^17^

Tumor necrosis factor receptor (TNFR)–associated factor 1 (TRAF1) is a signaling adaptor protein that has a multifaceted role in regulating the activation of NF-κB across different immune cell types and receptor signaling pathways. TRAF1 recruits the cellular inhibitor of apoptosis protein 2 (cIAP2) to promote signaling downstream of certain TNFR superfamily members while, on the contrary, TRAF1 interacts with the linear ubiquitin assembly complex (LUBAC) to restrain linear ubiquitination of key signaling proteins downstream of the Toll-like receptor (TLR) to dampen the inflammatory response.^18–21^ In a recent study, our group has identified a critical mutation in TRAF1 (V203A in humans; V196A in mice) that disrupts its interaction with cIAP2, resulting in a substantial reduction in inflammation, notable protection from LPS-induced septic shock, and a significant decrease in joint inflammation and disease severity in a model of rheumatoid arthritis (RA).^22^

We have previously shown that TRAF1 knockout mice were more susceptible to an inflammasome-driven model of MSU crystal–induced arthritis than TRAF1 wild-type (WT) littermates.^4^ Mechanistically, we showed that TRAF1 negatively regulates inflammasome activation in vitro and in vivo by attenuating the linear ubiquitination of the adapter protein ASC. Interestingly, cIAPs have been linked to mediating inflammasome formation and efficient caspase-1 activation through K63-linked polyubiquitination of caspase-1.^23^ We have recently identified a crucial role of TRAF1 in protecting cIAP2 from cIAP1-mediated degradation.^22^ Therefore, it follows that the TRAF1/cIAP2 axis could be involved in inflammasome formation and efficient caspase-1 activation. We hypothesized that abrogating the TRAF1/cIAP2 interaction could lead to reduced inflammasome activation and decreased K63-linked polyubiquitination of caspase-1. Here, we show that disrupting the TRAF1/cIAP2 interaction in THP-1–derived macrophages dampens NLRP3 inflammasome activation as a result of reduced K63-linked ubiquitination of caspase-1. Remarkably, mice harboring the TRAF1 V196A mutation produced significantly lower IL-1β levels in response to a sublethal dose of LPS and exhibited reduced joint inflammation and cellular infiltration in the MSU crystal–induced arthritis model compared with TRAF1 WT littermate mice.

## Materials and methods

### Cell culture and reagents

Primary bone marrow–derived macrophages (BMDMs) were prepared from C57BL/6 and TRAF1^V196A^ mice, as previously reported.^18^^,^^22^ THP-1 cells were obtained from the American Type Culture Collection (Manassas, VA, USA), and TRAF1^V203A^ THP-1 clones (C1 and C2) were generated through CRISPR/Cas9 editing of TRAF1, as previously described.^22^ All cells were cultured in RPMI 1640 media (MilliporeSigma, St Louis, MO, USA) supplemented with 10% heat-inactivated fetal bovine serum, 0.1% 2-mercapthoethanol, 1% l-glutamine–pyruvate–penicillin-streptomycin, and nonessential amino acids. LPS from *Escherichia coli* (serotype O55:B5; MilliporeSigma) was used for all in vivo and in vitro experiments.

### Mice and models of sepsis and MSU crystal–induced gout and sepsis

TRAF1^V196A^ mice were generated in the C57BL/6 background at the Toronto Centre for Phenogenomics (Toronto, ON, Canada), as previously described.^22^ C57BL/6 WT mice (Charles River Laboratories, Wilmington, MA, USA) were crossed with TRAF1^V196A^ mice and F1 progeny were bred for all in vivo experiments. Female littermate mice between 6 and 8 weeks old were used for the in vivo LPS challenge. LPS from *E. coli* (serotype O55:B5; MilliporeSigma) was diluted in sterile PBS and injected intraperitoneally (final volume 200 µL) into mice according to the body weight for a sublethal dose of 10 mg/kg. Serum was collected at various timepoints indicated in the figure legends to assess cytokine levels of IL-1β using the LEGENDplex bead-based flow cytometry immunoassay (BioLegend, San Diego, CA, USA). Male and female littermate mice between 10 and 12 weeks old were used for the MSU crystal-induced gout model. Mice received intra-articular injections into the knee joint with either PBS or 0.5 mg MSU crystals, administered into contralateral sides.^4^ Knee thickness was measured and after 4 hours, mice were euthanized, synovial tissues were dissected, and knee joints were fixed and stained for histology with hematoxylin (MilliporeSigma) and eosin (Thermo Fisher Scientific; Waltham, MA, USA), as previously described.^4^^,^^24^ All animals were housed under specific pathogen–free conditions at the Farquharson building vivarium at York University and all procedures were approved by the York University Animal Care Committee in accordance with Canadian Council on Animal Care regulations (protocol numbers 2019-12 for MSU Arthritis and 2023-06 for LPS Sepsis).

### Preparation of MSU crystals

MSU crystals were prepared by dissolving 1 g uric acid (MilliporeSigma) in 200 mL boiling double-distilled water containing 1 N NaOH (pH 7.2). Endotoxins were removed by autoclaving the solution for 6 hours at 120 °C. The limulus amebocyte lysate assay (Thermo Fisher Scientific) confirmed that the MSU crystals were free from detectable endotoxin contamination (<0.02 endotoxin U/mL). MSU crystals were assessed for appropriate crystal shape and birefringence before and after autoclaving.

### Co-immunoprecipitation and immunoblotting

Proteins in the culture supernatants collected from inflammasome-stimulated THP-1 cells were precipitated with 100% trichloroacetic acid (TCA) and then immunoblotted with antibodies to detect human/mouse IL-1β ELISA (R&D Systems, Minneapolis, MN, USA) and mouse caspase-1 (BioLegend). For K63 ubiquitin immunoprecipitations, whole cell lysates were incubated with Protein A/G beads (Pierce) and 2.5 µg of cleaved caspase-1 antibody (D57A2), as previously described.^23^ Samples were then immunoblotted by 4% to 15% SDS-PAGE gradient gels and immunoblotted with anti-Lys63 antibody (1:1,000 dilution; Millipore).

### Inflammasome assay and IL-1β ELISA

Primary BMDMs were primed with 100 ng/mL LPS (serotype O55:B5; MilliporeSigma) for 3 hours. At the end of the 3-hour treatment, the media was replaced with 1 mL of Opti-MEM reduced serum media (MilliporeSigma) and the cells were treated for 1 and 3 hours with either 5 mM ATP or 10 µM nigericin (signal 2). The samples were harvested, and the supernatant was collected to perform IL-1β ELISA according to the manufacturer’s instructions (Thermo Fisher Scientific). The absorbance was then measured using a plate reader (Varioskan; Thermo Fisher Scientific).

### Statistical analyses

All statistical analyses were performed with GraphPad Prism software. A 2-way analysis of variance (ANOVA) was used for comparison of multiple groups, with the multiple comparisons test indicated in the figure legends. For the histopathological evaluation, differences in the average inflammatory infiltrates per section were analyzed by unpaired *t*-test. The results were expressed as mean ± SEM with *P* values as indicated in the figure legends.

## Results

### Disrupting TRAF1/cIAP2 interaction suppresses inflammasome activation in human THP-1–derived macrophages

**Figure 1.**
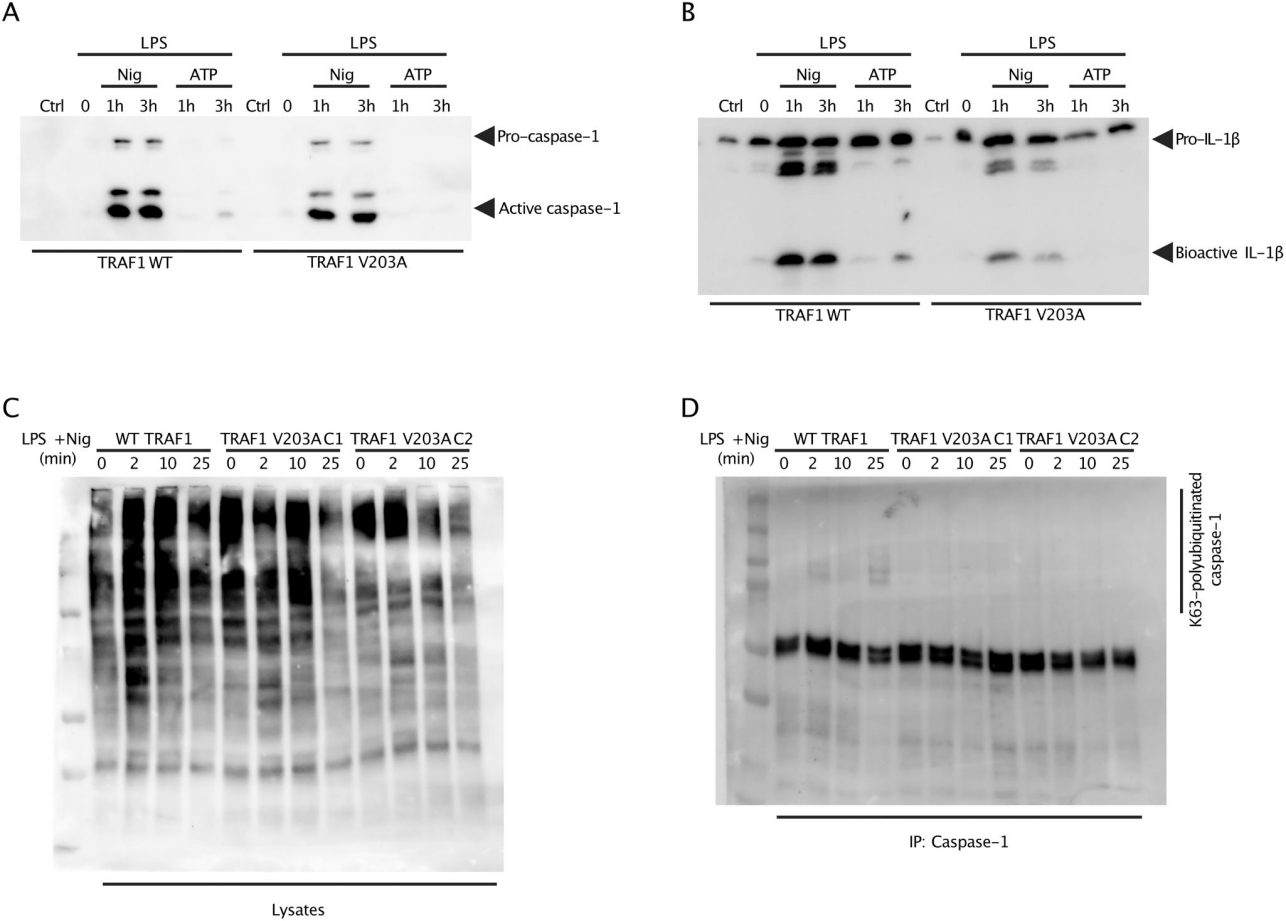
TRAF1 V203A mutation in THP-1 cells reduces secretion of caspase-1 and IL-1β by reducing K63-linked polyubiquitination of caspase-1. TRAF1 WT and TRAF1^V203A^ THP-1 cells were primed with or without 100 ng/mL LPS for 3 hours. Cells were then stimulated with either 5 mM ATP or stimulated with 10 µM nigericin for 1 hour (2 hours after priming step) and 3 hours (immediately after priming step). The culture supernatants were collected, and the proteins were precipitated using 100% TCA. (A) Supernatants were immunoblotted for immature caspase-1 (pro-caspase-1) and active caspase-1 (p20 subunit). (B) Supernatants were immunoblotted for inactive IL-1β (pro-IL-1β) and processed IL-1β (bioactive IL-1β). The blots shown in (A) and (B) are representative of five independent experiments with two of the identified clones containing the successful TRAF1 V203A mutation. (C) TRAF1 WT cells and TRAF1^V203A^ THP-1 clones (C1 and C2) were primed with or without 100 ng/mL LPS for 24 hours. Cells were stimulated with 10 µM nigericin for 2, 10, and 25 minutes. Whole-cell lysates were immunoblotted for K63-linked polyubiquitin (Ub) chains. (D) The whole-cell lysates from (C) were immunoprecipitated with endogenous caspase-1 and then immunoblotted for K63-linked polyubiquitin (Ub) chains. The blots shown in (C) and (D) are representative of two independent experiments with two of the identified clones containing the successful TRAF1 V203A mutation.

TRAF1 has been shown to interfere with the linear ubiquitination of ASC and thereby restricts NLRP3 inflammasome activation.^4^ However, TRAF1 can also recruit cIAP2 to TLR^22^ and TNFR^20^ signaling complexes to promote downstream NF-κB and mitogen-activated protein kinase (MAPK) signaling. To determine whether the specific disruption of the interaction between TRAF1 and cIAP2 affects inflammasome activation, we activated the NLRP3 inflammasome in THP-1 cells that had undergone CRISPR/Cas9 homology-directed repair–mediated knock-in of the TRAF1 V203A mutation.^22^ Interestingly, assaying the supernatant revealed that the TRAF1^V203A^ mutant THP-1 cells had decreased processing and secretion of both IL-1β and caspase-1 compared to WT THP-1 cells (Fig. 1A, B). Notably, there was no significant change in intracellular inactive forms of IL-1β or caspase-1 between WT and TRAF1^V203A^ mutant THP-1 cells (Fig. S1A). This indicates that disrupting the interaction between TRAF1 and cIAP2 downregulates the activation of the NLRP3 inflammasome.

### K63-linked polyubiquitination of caspase-1 is reduced in TRAF1^V203A^ THP-1 cells

Previous studies have shown that the TRAF1/cIAP2 interaction enhanced the stability of cIAP2 and protected cIAP2 from cIAP1-mediated degradation.^22^ Importantly, cIAP2, along with cIAP1, is required for optimal inflammasome formation and is essential for efficient caspase-1 activation through their nondegradative K63-linked polyubiquitination that is mediated by E3 ligase activity.^23^ Therefore, to understand why TRAF1^V203A^ THP-1 cells had lower inflammasome activation, we asked whether the disruption of the TRAF1/cIAP2 interaction impacted K63-linked polyubiquitination of caspase-1. Indeed, we demonstrated that the TRAF1^V203A^ mutant THP-1 cells (clones C1 and C2) had decreased levels of K63-linked polyubiquitination compared to TRAF1 WT THP-1 cells (Fig. 1C). Consistently, K63-linked polyubiquitination of caspase-1 was markedly reduced in TRAF1^V203A^ mutant clones at all timepoints compared to WT controls (Fig. 1D; Fig. S1B). This indicates that the TRAF1/cIAP2 interaction is important for efficient caspase-1 activation and inflammasome formation.

### TRAF1^V196A^ mice exhibit lower IL-1β secretion in vitro and in vivo

**Figure 2.**
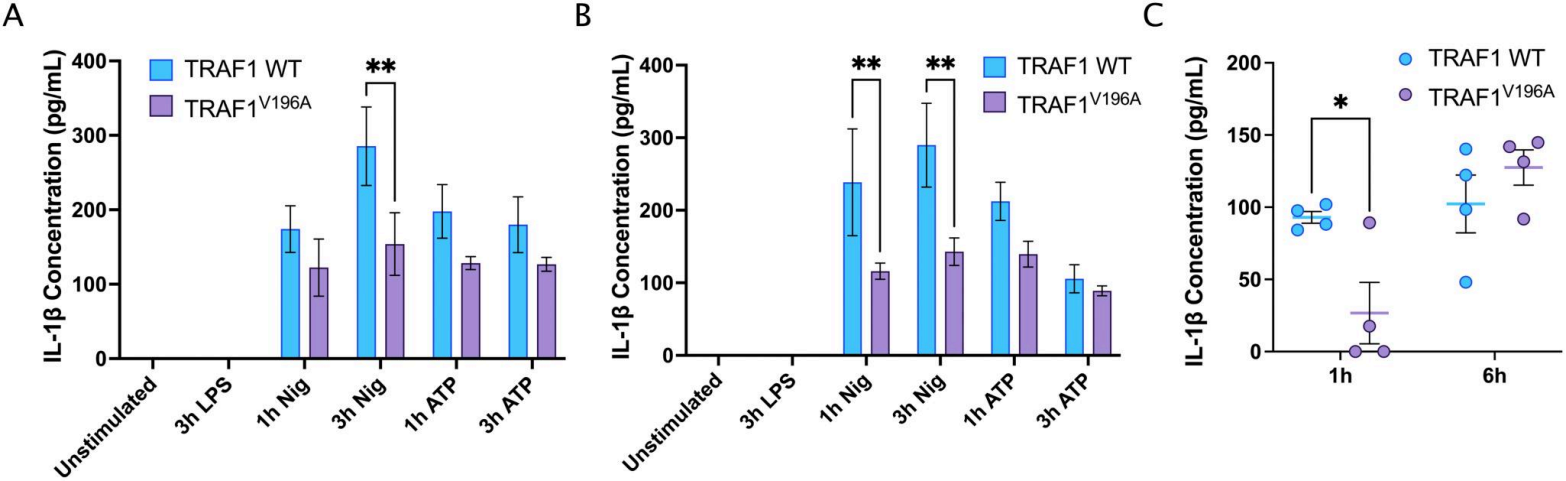
TRAF1^V196A^ mice exhibit reduced inflammasome-driven IL-1β secretion in vivo and in vitro. TRAF1 WT and TRAF1^V196A^ BMDMs were primed with or without 100 ng/mL LPS for 3 hours. Cells were then stimulated with either 5 mM ATP or stimulated with 10 µM nigericin for 1 hour (2 hours after priming step) and 3 hours (immediately after priming step). IL-1β was quantified from the collected supernatant by ELISA. A total of six independent experiments were performed, three in female mice (A) and three in male mice (B). Ordinary 2-way ANOVA was performed with Tukey multiple comparisons test to determine statistical significance. (C) TRAF1 WT (*n* = 4) and TRAF1^V196A^ (*n* = 4) female littermate mice were injected intraperitoneally with a sublethal LPS dose of 10 mg/kg. Blood was collected at the 1- and 6-hour timepoints by saphenous vein and the serum was collected to perform the multiplex cytokine assay by flow cytometry. Each symbol represents one mouse. Ordinary 2-way ANOVA was performed with Sidak multiple comparisons test to determine statistical significance. The error bars represent mean ± SEM. *P < 0.05, **P < 0.01.

We next asked whether disrupting the TRAF1/cIAP2 signaling axis would reduce inflammasome activation in vivo. A recent study from our group has shown that the V196A mutation in mouse TRAF1 leads to abrogation of its interaction with cIAP2 in BMDMs and reduced responses to LPS in in vivo models of sepsis and RA.^22^ To this end, we activated the NLRP3 inflammasome in BMDMs prepared from WT and TRAF1^V196A^ littermate mice. Strikingly, we show that IL-1β secretion in the supernatant following stimulation with LPS + nigericin was significantly reduced in both male and female TRAF1^V196A^ BMDMs compared to age- and sex-matched TRAF1 WT BMDMs (Fig. 2A, B). Notably, TRAF1^V196A^ BMDMs stimulated with LPS + ATP showed a slight reduction compared to WT controls, albeit statistically not significant. This could be attributed to the concentration and timing of ATP stimulation, which was manifested in the lower levels of IL-1β produced in WT and TRAF1^V196A^ BMDMs.

Unlike in BMDMs, where two signals are required to trigger the release of IL-1β from cells (eg LPS + nigericin), LPS alone is sufficient to promote the production of mature IL-1β in vivo since injured cells could release DAMPs, which serve as the second signal.^25–28^ Therefore, we asked whether TRAF1/cIAP2 interaction is needed for optimal IL-1β secretion following a sublethal challenge with LPS in mice. Remarkably, TRAF1^V196A^ mice exhibited lower levels of serum IL-1β compared to age- and sex-matched WT littermates at the 1-hour timepoint (Fig. 2C). These results demonstrate that disruption of the TRAF1/cIAP2 axis reduces the early (acute) IL-1β production in vivo, likely due to reduced cIAP2-mediated K63-linked polyubiquitination of caspase-1, as we showed in THP-1–derived macrophages. Notably, there was an increase in serum IL-1β in TRAF1^V196A^ mice at the 6-hour timepoint, suggesting the involvement of inflammasome-independent mechanisms^29–31^ in driving IL-1β production during the later stages of the systemic inflammatory response.

### TRAF1^V196A^ mice are less susceptible to MSU crystal–induced gout

**Figure 3.**
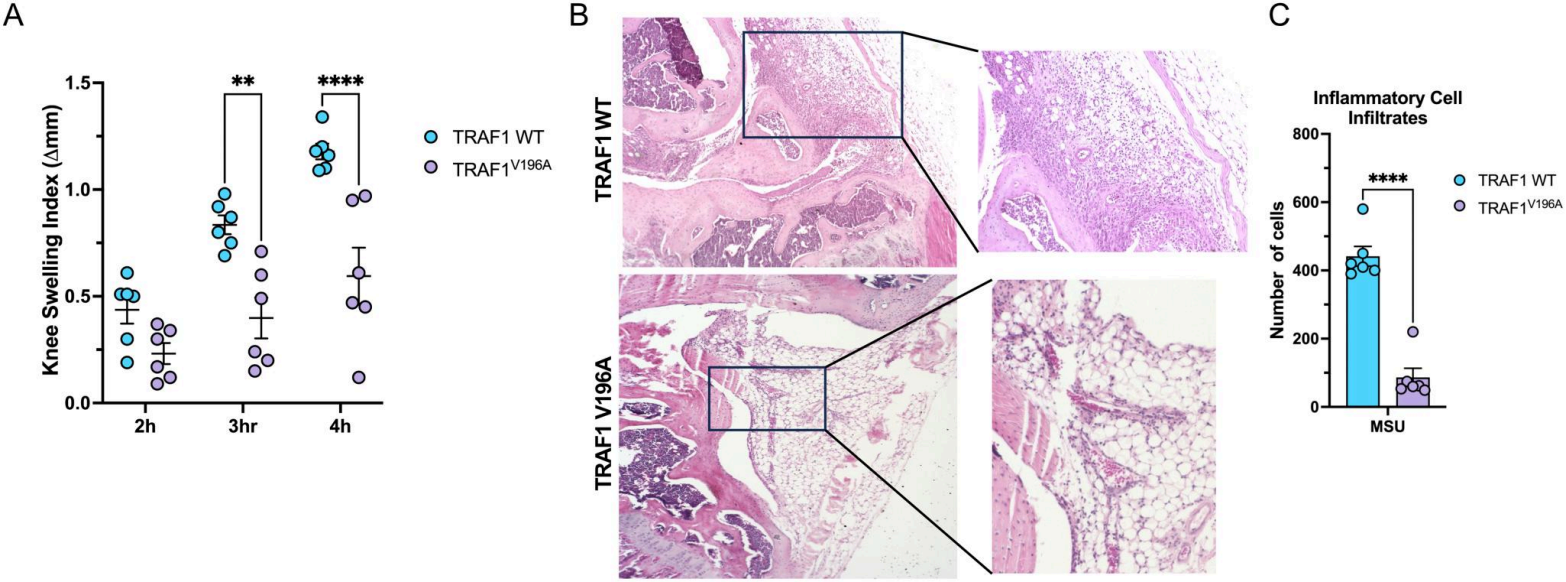
TRAF1^V196A^ mice exhibit reduced joint swelling and cellular infiltration following monosodium urate (MSU) crystal–induced arthritis. TRAF1 WT (*n* = 3 female, *n* = 3 male) and TRAF1^V196A^ (*n* = 3 female, *n* = 3 male) littermate mice were injected intraarticularly with 0.5 mg MSU crystals in one knee joint and PBS, as a control, in the contralateral knee joint. (A) Knee joint thickness was measured with a digital caliper before injection, then at 2, 3, and 4 hours following MSU injections. Data are reported as the change in thickness (Δmm). (B) Representative H&E staining images for TRAF1 WT and TRAF1^V196A^ littermate mice highlighting the histopathological differences in the synovial tissue of the knee joint following MSU crystal–induced arthritis. The results shown are representative images with at least 6 mice in each genotype. Ordinary 2-way ANOVA was performed with Sidak multiple comparisons test to determine statistical significance. (C) Quantification of the average inflammatory infiltrates per section, as shown by the histopathological evaluation in (B). Unpaired *t*-test was performed to determine statistical significance. The error bars represent mean ± SEM. **P < 0.01, ****P < 0.0001.

Research that uncovered TRAF1’s multifaceted role in regulating various signaling pathways in innate and adaptive immune cells has shed the light on its role in inflammatory arthritic diseases, including RA^22^^,^^32^ and gout.^4^ This is highlighted by numerous genome-wide association studies identifying TRAF1 single-nucleotide polymorphisms as risk factors for developing RA and for predicting the clinical response to anti-TNF therapy.^33^ Moreover, a recent study showed that TRAF1’s ability to negatively regulate the linear ubiquitination of ASC is crucial for controlling inflammasome activation and that TRAF1-deficient mice exhibited exacerbated inflammation and joint swelling in the MSU crystal–induced arthritis model, an NLRP3 inflammasome–dependent model of gout, compared to WT littermates.^4^ In contrast, TRAF1’s ability to stabilize and recruit cIAP2 to immune signaling platforms is necessary for optimal NF-κB and MAPK activation and a mutation that disrupts the TRAF1/cIAP2 interaction reduces cIAP2 levels and lowers joint inflammation in a model of RA.^22^ Thus, we asked whether the reduced inflammasome activation and IL-1β production observed in TRAF1 mutants that do not interact with cIAP2 could be beneficial in an inflammasome-driven disease like gout. Remarkably, TRAF1^V196A^ mice exhibited significantly reduced knee swelling compared to WT littermates following intra-articular injections of MSU crystals (Fig. 3A). Notably, the synovial and periarticular tissues appeared more intact, with less bony erosions and edema, in the knee joints of TRAF1^V196A^ mice compared to those in WT littermates (Fig. 3B). Moreover, inflammatory cell infiltrates in the synovium of the knee joints of TRAF1^V196A^ mice were also markedly lower than those in the WT littermates (Fig. 3B, C). These results demonstrate that the TRAF1^V196A^ mutation limits inflammation and joint swelling in a model of MSU crystal–induced gout.

Collectively, our results highlight the critical role of the TRAF1/cIAP2 interaction in regulating inflammasome activation. We demonstrated that disrupting the TRAF1/cIAP2 interaction dampens inflammasome activation by reducing K63-linked polyubiquitination of caspase-1, thereby decreasing IL-1β production. We also challenged mice with a sublethal dose of LPS and confirmed that the TRAF1^V196A^ mutation reduces IL-1β cytokine production in vivo. Finally, we employed the MSU crystal–induced arthritis model and showed that TRAF1^V196A^ mice exhibit significantly reduced inflammation and joint swelling compared to TRAF1 WT littermate mice. Ultimately, selectively targeting the TRAF1/cIAP2 interaction could pave the way for novel therapeutic strategies for inflammasome-driven diseases such as gout.

## Supplementary Material

vlaf065_Supplementary_Data

## Data Availability

The data that support the findings of this study are included in the article. Further inquiries can be directed to the corresponding author.
