# Supplementary material for: Disrupting the TRAF1/cIAP2 interaction attenuates inflammasome activation and protects against monosodium urate crystal–induced arthritis
Source: Immunohorizons. 2025 Nov 24;9(12):vlaf065. doi: 10.1093/immhor/vlaf065 (PMC12643475; doi:10.1093/immhor/vlaf065)
Supplement: vlaf065_Supplementary_Data [file vlaf065_supplementary_data.docx]

**
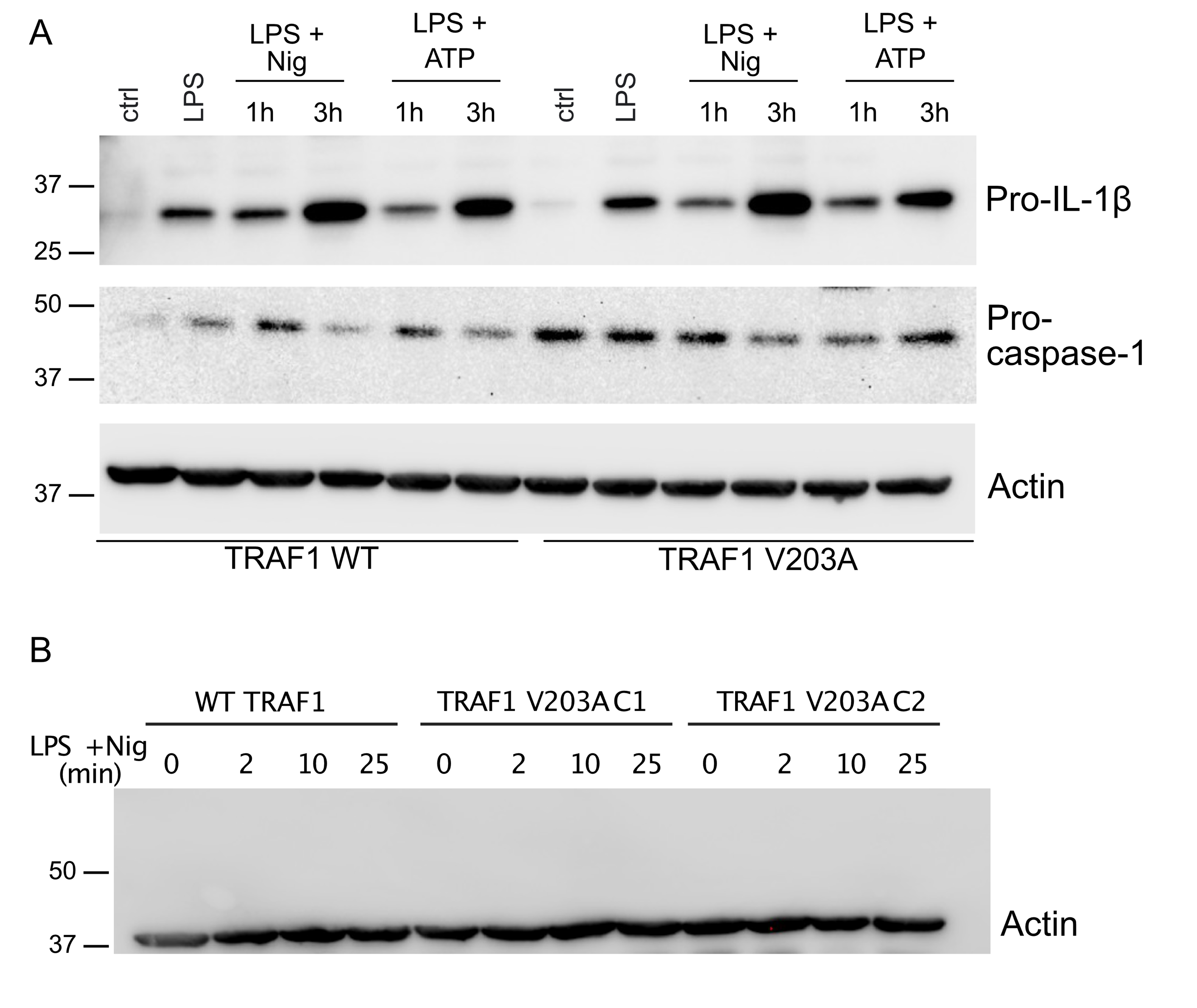
Figure S1. TRAF1 V203A mutation in THP-1 cells does not affect intracellular levels of pro-caspase-1 and pro-IL-1β.** (**A**) TRAF1 WT and TRAF1^V203A^ THP-1 cells were primed with or without 100 ng/mL LPS for 3 hours. Cells were then stimulated with either 5 mM ATP or stimulated with 10 μM nigericin for 1 hour (2 hours after priming step) and 3 hours (immediately after priming step). Whole cell extracts were then immunoblotted for caspase-1, IL-1β and actin, as a loading control. (**B**) TRAF1 WT cells and TRAF1V203A THP-1 clones (C1 and C2) from figure 1C were primed with or without 100 ng/mL LPS for 24 hours. Cells were stimulated with 10 μM nigericin for 2-, 10- and 25-minutes. Whole-cell lysates were immunoblotted for actin as a loading control.
